# Protective Effects of Specneuzhenide on Renal Injury in Rats with Diabetic Nephropathy

**DOI:** 10.1515/med-2019-0085

**Published:** 2019-10-06

**Authors:** Jiangning Yin, Jun Jiang, Huajun Wang, Guoyuan Lu

**Affiliations:** 1 Urology Department,No. 188, Shizi Street, Urology Department, The First Affiliated Hospital of Soochow University, Suzhou 213006, China; 2 Department of Emergency, Affiliated Hospital of Jiangsu University, Zhenjiang 212000, China; 3 Traditional Chinese Medicine and Pharmacy Department, School of Pharmacy Jiang Su University, Jiang Su 212013, China; 4 Pharmaceutical Department, Affiliated Hospital of Jiangsu University, Zhenjiang 212000, China

**Keywords:** Specnuezhenide, Diabetic nephropathy, Renal injury, Podocyte, Slit diaphragm protein, Inflammation

## Abstract

**Background:**

We aim to investigate the protective effects and potential mechanisms in specneuzhenide (SPE) on renal injury in rats with diabetic nephropathy (DN).

**Results:**

SPE could inhibit the decrease of body weight compared with the model group (P<0.05), and trigger improvement in the renal index (P<0.05). High dose and low dose SPE could trigger a significant decrease in serum IL1β, IL-6 and TNF-α compared with the model group (P<0.05). SPE could attenuate the glomerular lesions in DN rats. SPE induced up-regulation of podocin and CD2AP (P<0.05).

**Conclusion:**

SPE showed protective effects on renal injury through attenuating the pathological injury and urine protein. This process may be closely related to the modulation of CD2AP and podocin expression.

## 1 Introduction

Diabetic nephropathy (DN), a common late-stage complication of diabetes mellitus, is one of the leading causes for end-stage renal disease [1, 2]. Nowadays, the incidence of DN is on an increasing trend. According to a recent survey, more than 100 million patients are suffering from DN [3]. To our best knowledge, the pathogenesis of DN is rather complex, involving genetic, metabolic, hemodynamic, oxidative stress and inflammatory factors. Nowadays, increasing interest has been given to the roles of podocyte injury. For example, the slit membrane serving as the last barrier for glomerular filtration, plays important roles in the generation of proteins [4]. The nephrin/ CD2AP/podocin complex is a key functional unit for the slit membrane. Nowadays, there are still lacking appropriate treatment options for DN, and most of the treatment options utilize the reduction of glucose, regulation of blood fat, and application of angiotensin converting enzyme inhibitor (ACEI) and angiotensin-receptor blocker (ARB) agents. Extensive studies have revealed that some active components of traditional Chinese medicine (TCM) could regulate glycometabolism and oxidative stress as these components are efficient in prevention and treatment of DN [5, 6, 7].

Specneuzhenide (SPE), an isomer of oleanolic acid, is extensively prevalent in herbal medicine and natural drugs. It has been reported to be involved in the anti-oxidation, anti-inflammation and decline of blood glucose [8]. Besides this, SPE has been approved to inhibit the expression of NF-κB in diabetic rats, which then inhibited the inflammatory reaction in the vascular tissues in the presence of diabetes [9, 10]. Moreover, SPE contributed to urine protein excretion and renal tissue pathological changes in diabetic rats. Furthermore, it may show protective effects on the kidneys through decreasing the tumor necrosis factor-α (TNF-α) and IL-6 that may inhibit the inflammation in renal tissues [11, 12]. In a previous study, SPE induced improvement of sugar tolerance and insulin resistance in KKAy mice, which may be associated with the elevation of GLUT4 protein transmission and expression in muscle cells.

In this study, a DN model was induced through Streptozotocin (STZ) and a high glucose/high fat diet. Then we investigated the effects of SPE of various concentrations on the pathogenesis of DN. We aim to investigate the protective effects of SPE on the pathogenesis of DN.

## 2 Methods

### 2.1 Animals

Male Sparague Dawely (SD) rats (specific pathogen free; weighing 220±20 g; approval No.: SYXK20130036) were provided by the Animal Center of Jiangsu University. The animals were housed in separated cages in a 12-h light/12-h dark cycle at a temperature of 23°C with a humidity of 40%. The animals were on a normal diet and had free access to food and water. All animal experimental protocols were carried out in compliance with the Ethics Committee of the Affiliated Hospital of Jiangsu University.

### 2.2 Establishing the DN model

The DN model was induced using a low dose of STZ and a high glucose-high fat diet according to the previous description [13]. Briefly, rats were fed on a diet containing high glucose and high fat for 4 weeks, followed by fasting for 12 hrs. STZ (1%, 60 mg/kg) was given via intraperitoneal injection. Blood sample collection was performed via the caudal vein to determine the blood glucose about 72 hrs after STZ treatment. A glucose level of ≥16.7 mmol/L was considered as DM.

### 2.3 Grouping

Thirty-six DN rats were randomly divided into the following groups: (i) model group (n=12) subject to distilled water via lavage per day, (ii) low dose SPE group, subject to SPE injection via lavage per day (30 mg/kg), and (iii) high dose SPE group, subject to SPE injection via lavage per day (60 mg/kg). The administration duration was 16 days in each group. Twelve normal mice with no DN induction were subjected to distilled water via lavage served as control.

### 2.4 Determination of blood glucose and urine protein

Blood glucose was determined after sample collection from the caudal veins on week 2, 5 and 8 after SPE treatment in high and low doses groups, or distilled water treatment in the model and control groups. Blood glucose was determined using a glucose device (Onetouch, Johnson & Johnson) according to the manufacturer’s instructions. The 24 h urine protein was determined using the commercial kit purchased from the Jiancheng Biotech (Category No. C035-2, Nanjing, China), according to the manufacturer’s instructions.

### 2.5 Evaluation of inflammatory factors, SOD, MDA and renal index

For the determination of inflammatory factors in serum and kidney, the blood samples (1.5 mL) were collected from the orbit of each rat, followed by centrifugation at 5,000 rpm for 10 min to collect the serum. About 1 hr after the last treatment, all rats were sacrificed to obtain the renal tissues. The tissues were homogenated at 12,000 rpm for 10 min to obtain the supernatant. The inflammatory factors including IL-1β, IL-6 and TNF-α were determined using ELISA kits purchased from Jiancheng Biotech (Nanjing, China).

To determine the SOD and MDA, the renal tissues were homogenated at 5,000 rpm at 4°C for 15 min to obtain the supernatant. Then the SOD and MDA was measured using the commercial kits purchased from Jiancheng Biotech (Nanjing, China), respectively.

To calculate the renal index, the kidneys were obtained, followed by drying the surface using a filter paper. Then the weight was determined, and the renal index was calculated using the following formula: renal index= renal weight/total body weight× 100 %.

### 2.6 HE and PAS staining

The renal cortex was fixed using 10% formaldehyde for 24 hrs, followed by dehydration in 70%, 80%, 90%, 95% and 100% ethanols for 30 min. Then the samples were embedded in paraffin. The sections (2 μm) were then subject to HE staining and PAS staining according to the previous description (Al-Amiri, Chatrath, Bhawan, & Stefanato, 2003; Fischer, Jacobson, Rose, & Zeller, 2008). The pathological changes were observed under a light microscope.

### 2.7 Western blot analysis

Total RNA was extracted from the renal tissues (60 mg) using RIPA lysis. Protein concentration was determined using a Bradord protein commercial kit. Protein (30 μg) was separated by 10% SDS-PAGE, and then the gel was transferred to a PVDF membrane. Membranes were blocked with 5% (w/v) milk in TBS-T buffer, followed by incubating with the rabbit anti-rat podocin antibody (1:1000, Abcam, CA, USA) and rabbit anti-rat CD2AP antibody (1:300, Abcam, CA, USA). The membrane was then incubated with HRP-conjugated secondary antibodies for 1h. The same membrane was probed for β-actin for loading control. The gray value was analyzed with the Quantity one v4.62 software (Bio-Rad, CA, USA).

### 2.8 Statistical analysis

All the data were presented as mean ± standard deviation. SPSS19.0 software was used for the statistical analysis. Analysis of variance was used for the comparison between groups. P<0.05 was considered to be statistically significant.

## 3 Results

### 3.1 Effects of SPE on glucose and urine protein in DN rats

#### 3.1.1 SPE induced decline of blood glucose

No statistical differences were noticed in the blood glucose at the baseline levels among the four groups (P>0.05, Table 1). The blood glucose in the model group showed a significant increase at week 4, 8, 12 and 16 after DM induction compared with the control group (P<0.05, Table 1). In the SPE-L group, the blood glucose showed a decrease at week 12 and 16 compared with the model group (P<0.05). In the SPE-H group, compared with the model group, a significant decrease was noticed at week 4, 8, 12 and 16, respectively (P<0.05). These indicated that the SPE contributed to the decline of blood glucose.

**Table 1:** Regulatory effects of SPE on glucose

Group	Baseline	Week 4	Week 8	Week 12	Week 16
Control	4.46 ± 0.02	4.49 ± 0.02	4.61 ± 0.08	4.51 ± 0.06	4.47 ± 0.03
Model group	4.48 ± 0.04	23.78 ± 0.21	25.53 ± 0.68	26.21 ± 0.62	25.30 ± 0.52*
SPE-L	4.41 ± 0.02	23.34 ± 0.97	21.32 ± 0.96	16.96 ± 0.82	15.77 ± 0.79^†^
SPE-H	4.45 ± 0.03	17.44 ± 0.68	12.78 ± 0.82	9.39 ± 0.64	8.46 ± 0.72^†^

* P < 0.05 versus control; ^†^P < 0.01 versus model group.

#### 3.1.2 SPE induced significant decrease of 24h urine protein

Compared with normal control, a significant increase was noticed in the 24 h urine protein at week 4, 8, 12 and 16, respectively (P<0.01). Compared with the model group, significant improvement was noticed in the urine protein in SPE groups. At week 16, significant decrease was noticed in the 24 h urine protein in high dose group and low dose group compared with that of the model group (P<0.05, Table 2).

**Table 2:** Comparison of 24 h urine protein in each group

Group	Baseline	Week 4	Week 8	Week 12	Week 16
Control	7.02 ± 0.63	7.68 ± 0.03	6.81 ± 0.16	6.42 ± 0.06	7.37 ± 0.23
Model group	7.37 ± 0.42	26.88 ± 0.49	28.44 ± 0.10	28.40 ± 0.25	32.64 ± 0.41*
SPE -L	7.28 ± 0.61	24.55 ± 0.83	26.88 ± 0.94	25.98 ± 0.81	25.01 ± 0.86 ^†^
SPE -H	7.14 ± 0.64	21.39 ± 0.72	23.47 ± 0.73	24.49 ±0.75	21.76 ± 0.95 ^†^

* P < 0.01 versus control; ^†^P < 0.05 versus model group.

#### 3.1.3 Effects of SPE on the body weight, renal weight and renal index in DM rats

Compared with the normal control, the body weight in the model group showed a significant decrease, especially the weight of kidney and renal index (P<0.01). In the SPE-H group, a significant increase was noticed in the body weight compared with the model group (P<0.05). Besides this, a high dose of SPE contributed to the decrease of kidney weight and renal index compared with the model group. In contrast, low dose of SPE induced no statistical differences in the body weight, kidney weight and renal index compared with the model group (P>0.05, Table 3).

**Table 3:** Effects of SPE on body weight, renal weight and index in DN rats

Group	Weight (g)	Renal weight (g)	Renal index (×100)
Control	406.32 ± 11.54	1.57 ± 0.04	0.38 ± 0.0045
Model group	327.83 ± 4.83 ^*^	1.74 ± 0.05 ^*^	0.53 ± 0.0201 ^*^
SPE-L	336.73 ± 3.09	1.66 ± 0.09	0.49 ± 0.0112
SPE-H	355.60 ± 5.19 ^†^	1.60 ± 0.12 ^†^	0.45 ± 0.0105 ^†^

* P < 0.05 versus control; ^†^P < 0.01 versus model group.

### 3.2 SPE modulated the expression of inflammatory factors in DN rats

#### 3.2.1 Effects of SPE on the inflammatory factors in serum and renal tissues in DM rats

Serum IL-1β showed significant elevation in the model group compared with that of the control group (P<0.05). Compared with the model group, a significant decrease was noticed in the IL-1β in SPE-H and SPE-L groups (P<0.05, Table 4).

**Table 4:** Regulatory effects of SPE on serum IL-1β, IL-6 and TNF-α

Group	Serum IL-1β (pg/mL)	Serum IL-6 (pg/mL)	Serum TNF-α (pg/mL)
Control	10.10 ± 2.18	20.64 ± 2.03	16.96 ± 5.23
Model group	35.06 ± 3.18 ^*^	40.10 ± 2.72 ^*^	41.27 ± 3.02 ^*^
SPE-L	27.45 ± 1.66 ^†^	36.05 ± 1.49	32.33 ± 5.84 ^†^
SPE-H	26.34 ± 1.32 ^#^	32.80 ± 1.52 ^†^	27.98 ± 4.25 ^#^

* P < 0.05 versus control; ^†^P < 0.05 versus model group; ^#^P < 0.01 versus model group.

Serum IL-6 in the model group showed significant elevation compared with that of normal control (P<0.05, Table 4). Compared with the model group, the serum IL-6 in the SPE-H group showed a significant decrease (P<0.05). Compared with the model group, no statistical differences were noticed in the serum IL-6 in the SPE-L group (P>0.05, Table 4).

The TNF-α expression in the model group showed significant elevation compared to the normal control (P<0.05). Compared with the model group, a significant decline of TNF-α in DM rats was noticed in the SPE-H group (P<0.01). Compared with the model group, TNF-α in the SPE-L group showed a significant decrease (P<0.05, Table 4).

#### 3.2.2 Effects of SPE on the IL-1β, IL-6 and TNF-α expression in renal tissues

Compared with the normal control, the level of IL-1β in renal tissues of DM rats showed significant increase in the model group. Nevertheless, compared with the model group, a significant decrease was noticed in IL-1β expression in renal tissues in the SPE-H and SPE-L groups, respectively (P<0.05, Table 5).

**Table 5:** Regulatory effects of SPE on renal IL-1β, IL-6, SOD, MDA, podocin and CD2AP

Group	Renal IL-1β (pg/mL)	Renal IL-6 (pg/mL)	Renal TNF-α (pg/mL)	Renal SOD (U/mL)	Renal MDA (nmol/mL)	Renal podocin	Renal CD2AP
Control	3.67 ± 0.37	6.80 ± 0.62	4.71 ± 0.89	22.57 ± 0.08	36.56 ± 9.26	0.70 ±0.03	0.73 ± 0.02
Model group	9.59 ± 1.37 *	9.71 ± 0.39 *	11.06 ± 0.32 *	9.09 ± 0.81 *	91.56 ± 8.56 *	0.06 ± 0.02 *	0.07 ± 0.03 *
SPE-L	7.37 ± 0.79 ^†^	9.65 ± 0.74	10.74 ± 0.86	16.43 ± 0.92 ^†^	67.48 ± 5.71 ^†^	0.21 ±0.03 †	0.19 ± 0.04 ^†^
SPE-H	5.55 ± 0.67 ^△^	7.23 ± 0.56 ^†^	8.17 ± 0.75 ^#^	22.31 ± 0.99 ^†^	53.68 ± 7.44 ^†^	0.51 ±0.05 3	0.49 ± 0.05 ^#^

* P < 0.05 versus control; ^†^P < 0.05 versus model group; ^#^P < 0.01 versus model group.

Compared with the normal control, a significant increase was noticed in IL-6 and TNF-α in renal tissues in model group (P<0.05, Table 5). IL-6 and TNF-α in renal tissues of DM rats showed significant decrease in SPE-H group compared with model group (P<0.05). In contrast, no statistical difference was noticed in the IL-6 and TNF-α in renal tissues of the SPE-L group compared with the model group (P>0.05, Table 5).

### 3.3 SPE affected the anti-oxidative stress in DN rats

#### 3.3.1 Effects of SPE on the anti-oxidant indices in renal tissues of DM rats

Compared with the normal control, the SOD activity in renal tissues of the model group showed a significant decrease (P<0.05, Table 5). Compared with the model group, the SOD activity in the SPE-L and SPE-H groups showed significant elevation. This implied that SPE induced satisfactory anti-oxidant capacity.

Compared with the normal control, the MDA content in renal tissues showed significant elevation in the model group (P<0.05). The MDA activity in the renal tissues in the SPE-H and SPE-L groups showed a significant increase compared with the model group (P<0.01, Table 5). These indicated that SPE played important roles in anti-oxidation and modulation of renal injury.

### 3.4 SPE showed protective effects on the podocyte injury in DN rats

#### 3.4.1 Expression of CD2AP and Podocin protein

SPE could obviously improve the expression of Podocin and CD2AP in the podocytes. Compared with the normal control, a significant decrease was observed in the expression of Podocin and CD2AP protein in the renal tissues in the model group (P<0.01). Compared with the model group, significant upregulation was noticed in the expression of Podocin and CD2AP protein in the renal tissues of the SPE-H group and SPE-L group, respectively (P<0.05, Table 5).

### 3.5 Protective effects of SPE on the renal function in DN rats

#### 3.5.1 HE staining results

**Figure 1:**
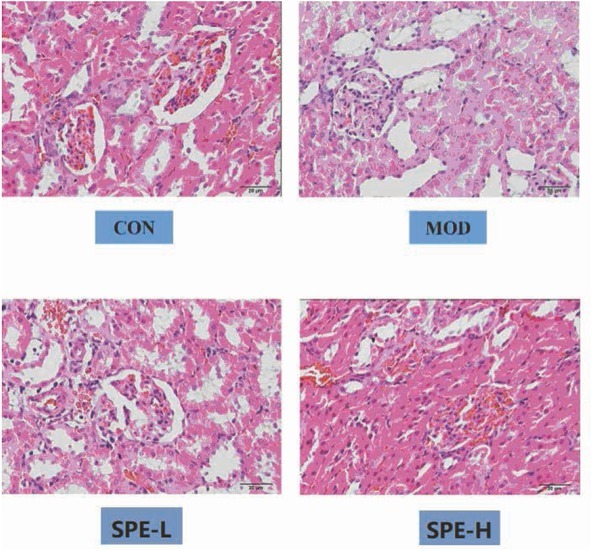
HE staining indicating the pathological changes in renal tissues. CON, control; MOD: model group; SPE-L, low dose group of SPE; SPE-H, high dose group of SPE.

For HE staining, the morphology of the kidney in the normal control was normal. The anatomical structure was clear. The morphology of the renal glomerulus was regular. No dilatation was noticed in the capillary lumen. The arrangement of the renal tubule was regular and tight. The morphology of epithelial cells in the renal tubule was normal. No atrophy or hypertrophy was noticed in the renal glomerulus. Basilar membrane and mesenterium showed no thickening. Compared with the normal control, the glomerular volume in the model group showed enlargement. Obvious thickening was observed in the capillary basement membrane. The matrix of the mesenterium showed obvious hyperplasia. Vacuolation was noticed in the renal tubular epithelial cells, in which protein was observed. In the SPE treatment groups, the pathological changes showed attenuation in DM rats to some extent. Besides this, the morphology of the renal glomerulus was normal. Part of the basilar membrane showed thickening. Slight hyperplasia was noticed in the mesenterium. Few renal tubular epithelial cells showed vacuolation. Compared with the model groups, significant attenuation was noticed in the pathological changes in the SPE groups (Figure 1).

#### 3.5.2 PAS staining results

**Figure 2:**
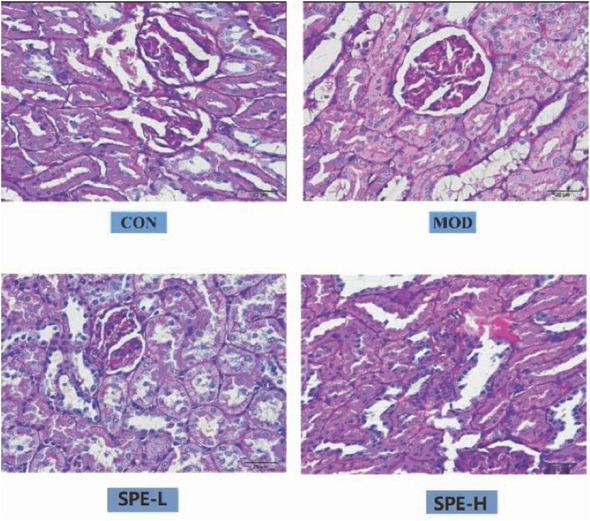
PAS staining for the pathological changes in renal tissues. CON, control; MOD: model group; SPE-L, low dose group of SPE; SPE-H, high dose group of SPE.

PAS staining results indicated that the ratio of area in renal tissues with positive staining showed significant elevation in the model group compared with the normal control. Compared with model group, the ratio showed significant decrease in the SPE groups, which were featured by inhibition of enlargement of renal glomerular volume, basilar membrane thickening and mesenteric hyperplasia (Figure 2).

## 4 Discussion

In this study, the DN model was induced by STZ and a high glucose or high fat diet. Then we determined the expression of inflammatory factors (e.g. IL-1β, IL-6 and TNF-α) and podocyte related protein (e.g. podocin and CD2AP), as well as the pathological changes in the kidney. We investigated the potential effects of SPE on the DN.

SPE could effectively attenuate the blood glucose and the expression of inflammatory factors in serum and renal tissues. Besides this, it could enhance the anti-oxidant capacity of renal tissues, which then contributed to the improvement of renal function and renal protection. According to the previous description, high glucose was involved in mediating the oxidative stress, which participated in the pathogenesis and development of DNA [14, 15]. Reactive oxygen species (ROS) accumulation was a crucial feature for DN. ROS induced the generation of lipid peroxidation products such as MDA, hydroxyl groups and ketone groups, which then led to renal injury by modulating the oxidation of amino acids and protein. As a type of antioxidase, SOD was reported to reflex the clearance of free radicals and the anti-oxidant capacity that may involve in the protection of cells from injury [16]. To our best knowledge, the decrease of anti-oxidant capacity was an important indicator for oxidative stress [17]. In this study, compared with the normal control, the MDA in renal tissues showed a significant increase (P<0.01), and SOD activity showed a significant decrease (P<0.01) in the model group. This implied that oxidative stress in renal tissues showed a significant increase in DN rats. However, after SPE treatment, blood glucose and MDA in renal tissues showed a significant decline compared with the model group. A significant elevation was also noticed in SOD activity. This implied that SPE showed satisfactory anti-oxidant capacity and brought down the blood glucose, which was in line with the previous descriptions.

For the pathogenesis of inflammatory injury in DN, elevation of oxidative stress and hemokinesis imbalance mediated the infiltration of white blood cells, macrophages and T-lymphocytes, which contributed to the release of inflammatory cells including IL-1, IL-6, IL-1β, TNF-α and INF-γ [18]. These inflammatory factors could further induce the generation of chemotactic factor in renal tissues, which then induced the infiltration of inflammatory cells. Finally, such process may trigger a severe injury in renal tissues. As an important factor for renal injury, TNF-α triggered the overexpression of various inflammatory factors and chemotactic factors (e.g. IL-6) [19]. Besides, it may enhance the inflammatory reactions. The generated IL-6 may induce the expression of factors associated with fibrosis, which then resulted in hypertrophy and fibrosis of renal tissues [20]. Moreover, overexpression of IL-6 in DN induced changes of permeability of the renal endothelium, which contributed to the expression of fibronectin and thickening of the glomerular basement membrane [21]. In this study, the basic structure injuries that induced inflammation and cellular infiltration were obvious in the renal tissues of DN rats, while the expression of TNF-α and IL-6 in the renal tissues showed a significant increase, which then induced the significant down-regulation of renal function. Administration of SPE (16-week) could obviously inhibit the infiltration of inflammatory cells, and down-regulate the IL-6 and TNF-α. These triggered the gradual improvement of the renal function. Therefore, we speculated that SPE could inhibit the inflammatory factors induced renal injury through inhibiting the expression of inflammatory factors such as TNF-α, IL-6 and IL-1β.

Podocytes, the highly differentiated terminal cells of glomerular basement membrane, are important components for the glomerular filtration barrier [22]. These cells have been reported to be closely related to the pathogenesis of DN. Besides, the podocytes-associated proteins were regarded to be associated with podocyte skeletal framework [23]. For instance, Podocin, CD2AP and nephrin could form a protein complex, which is then involved in the maintenance of the slit diaphragm and podocyte skeletal framework stability [24]. The expression of nephrin, Podocin and CD2AP was significantly down-regulated in the renal tissues of DN patients, which then resulted in the injury of the slit diaphragm and an increase of urine protein [25]. In this study, the expression of Podocin and CD2AP showed significant up-regulation in SPE groups compared with the model group. The urine protein also showed significant decrease after SPE treatment compared with the model group (P<0.05). On this basis, we speculated that SPE could up-regulate the expression of Podocin and CD2AP, which then led to stabilization of the slit diaphragm and protection of the renal function in DN rats.

Indeed, there are some limitations in this study. Although we revealed the protective effects of SPE on podocyte function, we did not present the morphological changes of these cells. In future, we will add such information, in order to further present the potential mechanisms in this process.

## 5 Conclusions

In summary, SPE contributed to the attenuation of urine protein content, which then obviously decreased the blood glucose in DN rats. Additionally, SPE could up-regulate the expression of CD2AP and Podocin in the slit membrane in the renal tissues of DN rats. This implied that SPE could up-regulate the Podocin and CD2AP expression, which could protect the renal function in DN rats. The protective effects of SPE on the attenuation of urine protein and protection of renal function were associated with the renal podocytes and the expression of CD2AP and Podocin in the slit membrane.

## Abbreviations

DN: diabetic nephropathy;
ROS: reactive oxygen species;
SD: sparague Dawely;
SPE: specneuzhenide;
STZ: streptozotocin;
TNF-α: tumor necrosis factor-α
